# Phemindole, a Synthetic Di-indole Derivative Maneuvers the Store Operated Calcium Entry (SOCE) to Induce Potent Anti-Carcinogenic Activity in Human Triple Negative Breast Cancer Cells

**DOI:** 10.3389/fphar.2016.00114

**Published:** 2016-05-04

**Authors:** Supriya Chakraborty, Swatilekha Ghosh, Bhaswati Banerjee, Abhishek Santra, Arghya Adhikary, Anup K. Misra, Parimal C. Sen

**Affiliations:** ^1^Division of Molecular Medicine, Bose InstituteKolkata, India; ^2^Centre for Research in Nanoscience and Nanotechnology, University of CalcuttaKolkata, India

**Keywords:** Phemindole, triple negative breast cancer (TNBC), stromal interaction molecule1 (STIM1), store-operated calcium entry (SOCE), human breast carcinoma MDAMB-231 cells

## Abstract

Triple-negative breast cancer (TNBC), is a specific subtype of epithelial breast tumors that are immuno-histochemically negative for the protein expression of the estrogen receptor (ER), the progesterone receptor (PR) and lack over expression/gene amplification of HER2. This subtype of breast cancers is highly metastatic, shows poor prognosis and hence represents an important clinical challenge to researchers worldwide. Thus alternative approaches of drug development for TNBC have gained utmost importance in the present times. Dietary indole and its derivatives have gained prominence as anti-cancer agents and new therapeutic approaches are being developed to target them against TNBC. But a major drawback with 3, 3′di Indolyl methane (DIM) is their poor bioavailability and high effective concentration against TNBC. However, the Aryl methyl ring substituted analogs of DIM display interesting anti-cancer activity in breast cancer cells. In the current study we report the synthesis of a novel synthetic aryl methyl ring substituted analog of DIM, named as Phemindole as an effective anti-tumor agent against TNBC cells. Furthermore, we enumerated that Phemindole caused reactive oxygen species mediated mitochondrial-dependent apoptosis in MDAMB-231 cells. Furthermore, Phemindole mediated Store Operated Calcium Entry (SOCE) retardation favored inactivation of STIM1 and henceforth activated ER stress to induce apoptosis in TNBC cells. Simultaneously, Phemindole was also found to restrict the *in vitro* cell migration through its anti mitotic property and pFAK regulation. Studies extended to *ex ovo* and *in vivo* mice models further validated the efficacy of Phemindole. Thus our results cumulatively propose Phemindole as a new chemotherapeutic regime which might be effective to target the deadly aspects of the TNBC.

## Introduction

Breast cancer is often not considered as a single disease but a family of diseases. Triple-negative breast cancer (TNBC), is a specific subtype of epithelial breast tumors that are immuno-histochemically negative for the protein expression of the estrogen receptor (ER), the progesterone receptor (PR) and lack over expression/gene amplification of HER2 (Kurose et al., 2001; Hayes, 2010). There are many treatment options for the hormone receptor “positive” tumors but fewer options exist for those that don’t. In fact no specific molecular targets for this subgroup of breast cancer patients are yet identified thus providing a major arena for exploration. Various reports suggest that this class of tumors are extremely aggressive in nature and have been found to show distant metastasis (De Laurentiis et al., 2010; Hudis and Gianni, 2011). To improve clinical prognosis of TNBC, we must elucidate the signaling pathways operating and the modes of development of TNBC and utilize this knowledge in improving the present day therapy regimes. Therefore, development of therapeutic strategies to target these TNBC cells may help to overcome the drawbacks in TNBC treatment.

Calcium (Ca^2+^) controls different cellular processes like short-term responses such as secretion and muscle contraction as well as long-term regulation of cell proliferation and differentiation (Berridge et al., 2000, 2003). Different mechanisms are involved to maintain the difference in calcium concentration from outer environment to cellular cytoplasm. Store-operated Ca^2+^ entry (SOCE) is one of the major mechanism by which cell maintains calcium homeostasis. In response to depletion of Ca^2+^ from intracellular Ca^2+^ stores [primarily the endoplasmic reticulum (ER)] cell activates specific plasma membrane channels, termed as store operated channels (SOCs) (Putney, 1986; Spassova et al., 2006). Stromal interacting molecule 1 (STIM1) is an ER- residential highly conserved type-I membrane, protein, containing a luminal EF-hand Ca^2+^-binding domain. It plays a dual role as an ER calcium concentration sensor and activator of SOCE (Liou et al., 2005; Spassova et al., 2006). STIM1 senses the depletion of Ca^2+^ from the lumen through its EF hand and initiates the process of store-operated Ca^2+^ entry, where a pool of STIM1 translocates to the plasma membrane to regulate SOCE, but the population of STIM1 that activates SOCE remains in the ER and co-clusters with Orai1 in ER-plasma membrane junctions (Putney, 1986; Liou et al., 2005; Spassova et al., 2006).

Recently various studies implicate that STIM and Orai proteins mediated SOCE is responsible in various processes during oncogenic transformation, apoptosis, proliferation, angiogenesis, metastasis and antitumor immunity. Ca^2+^ signaling mediated by SOCE is needed to induce genetic changes in normal cells, which implicates malignancies in premalignant cells. Several evidences indicate that STIM/ORAI-mediated SOCE promotes uncontrolled growth and metastasis in different types of cancer. McAndrew et al. (2011) showed from 295 breast cancer microarray data, that patients with a STIM1-high profile demonstrated abnormal SOCE and poorer prognosis. Orai1 and STIM1 expression were enhanced in therapy-resistant other types of carcinoma cells also (Xie et al., 2015). Pharmacological inhibition of STIM1- mediated SOCE can surprisingly enhance chemotherapy mediated lung or pancreatic cancer regression (Li W. et al., 2013; Kondratska et al., 2014). As a result, there has been an urgent need to develop of SOCE inhibitors that can be used to fight against cancer. Over the past decades, several small-molecule SOCE inhibitors have been developed which showed great promise in the chemotherapy. As SOCE implicates in several cell responses such as cell growth, survival, and apoptosis, it represent a primary target for treatment of TNBC also.

In chemotherapy natural product-oriented synthetic derivatives have played a significant role (Koehn and Carter, 2005; Mishra and Tiwari, 2011). The commonly used anti cancer agents such as paclitaxel and actinomycin C were originally developed from natural products. 3,3′-di-inolyl methane (DIM) produced in the stomach from indole-3-carbinol (I3C) which is the main ingredients of vegetables of the *Brassica* family. I3C is converted via acid-catalyzed reactions in the stomach in its most biologically active metabolite DIM (Bjeldanes et al., 1991). DIM has been studied extensively as an anticancer agent due to its ability to inhibit the growth of various type of cancer cell types *in vitro* and *in vivo* (Nachshon-Kedmi et al., 2004) and has showed promising results in clinical trials for the treatment of prostate cancer (Heath et al., 2010). Nevertheless, the development of DIM as a potent therapeutic agent is limited by numerous factors which are mainly because of its easy transformation into many polymeric products *in vivo* (Selvaraj et al., 2015). These compounds have some general targets but have some prominent biological effects on breast cancer cells and significantly high concentrations are required to arrest cell cycle progression in breast cancer cells (from 50 to 200 μM) (Safe et al., 2008). As alternatives to DIM as a chemotherapeutic agent for the treatment of breast cancer, several DIM analogs are now being characterized showing higher anti-proliferative properties (Dejeans et al., 2010; Li G. et al., 2013).

In the current study, we have reported the synthesis of a new DIM derivative Phemindole [3,3′-(4-hydroxyphenylmethylene)-bis-(7-methy-1H-indole)] and our experimental findings revealed that it exhibited better anti-tumor effect when directed against triple negative breast cancer (TNBC) cells than DIM alone. In this study, we showed that Phemindole exhibited *in vitro* potency that is two orders of magnitude higher than that of DIM in suppressing the proliferation of TNBC tumor cells. Furthermore, we have delineated the mechanistic role of Phemindole in inducing apoptosis in TNBC cells *in vitro* as well as tumor regression in *ex ovo* models respectively. It has been acknowledged that 4T1 cells are a murine TNBC cell line which serves as a suitable mouse model for the study of TNBC (Pan et al., 2012); therefore we also developed the 4T1 murine mammary carcinoma model in BALB/c mice and validated the effect of Phemindole in tumor regression *in vivo*. Underlying molecular mechanisms revealed that Phemindole showed regulation of cytochrome c via mitochondrial dependent pathway in TNBC cells. Simultaneously, Phemindole was found to regulate Store Operated Calcium Entry (SOCE) by down regulating STIM1 protein in TNBC cells. STIM1 played an important role in gene regulation by controlling the ER calcium homeostasis in breast cancer cells (Lax et al., 2006). Treatment of the TNBC cells with Phemindole favored down-regulation of the elevated STIM1 levels through retarded translocation from ER to the plasma membrane ORAI channel ultimately resulting in ER stress generation and apoptosis of TNBC cells. Our study thus reveals for the first time an intricate mechanism of how the Phemindole triggered death in highly aggressive TNBC cells. These results indicate the translational potential of Phemindole as a component of chemotherapeutic strategies for TNBC treatment.

## Materials and Methods

### Chemical Synthesis and Characterization of Phemindole

**FIGURE 1:**
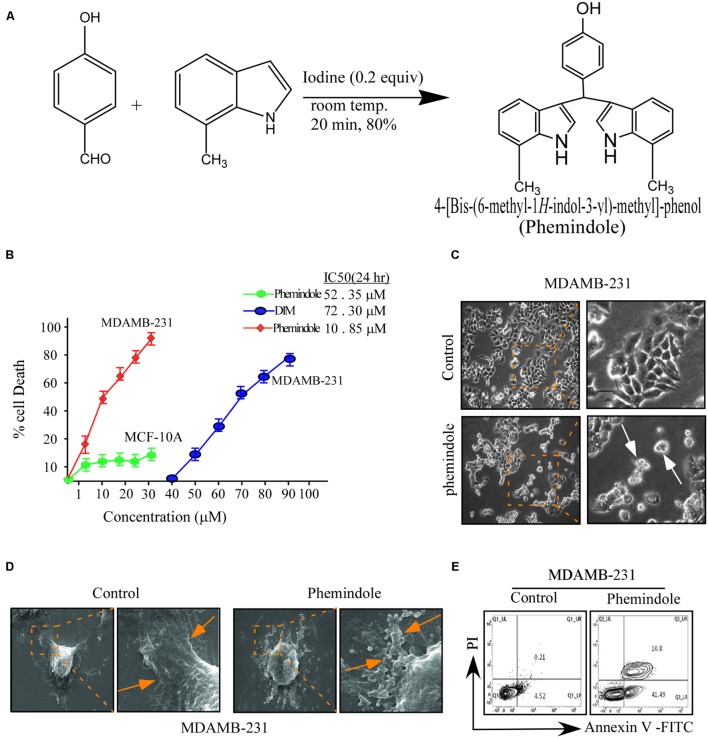
**Effects of di-indolyl methane analog, Phemindole on cell viability and apoptosis in human triple negative breast cancer cells.** **(A)** The chemical structure of di-indolyl methane and its analog, Phemindole. **(B)** The effect of Phemindole on cell viability in human breast cancer cells, MDAMB-231 and normal breast epithelia, MCF-10A. Cells were treated with Phemindole and DIM at different concentrations for 24 h as indicated cell viability was determined by MTT assay, IC50 values were indicated for each treatment. **(C)** Phase contrast images showing the effect of Phemindole (10 μM) on MDAMB-231 cells. Arrows in the treated sets are indicating apoptotic cells. **(D)** SEM micrographs of MDAMB-231 cells showing cellular membrane blabbing due to apoptosis in Phemindole treated set (indicated by arrows) (right). Untreated control cell showed normal morphology (left) **(E)** Representative images for cell apoptosis stained with Annexin V-FITC/PI. Cells were treated with Phemindole at 10 μM concentration for 24 h, followed by staining with Annexin V-FITC/PI and analyzed by flow cytometry as described in methods. Values are mean ± SE from three independent experiments. *p < 0.05, **p < 0.01; all versus control group.

On the basis of previous discovery on natural product analogs as anti cancer agent we have designed the anti cancer compound of 3,3′-(4-hydroxyphenylmethylene)-bis-(7-methy-1H-indole). We have been reviewing the Aryl methyl ring substituted analogs of 3,3′ di indolyl methane because there are many reports in the literature displaying interesting anti-cancer activity of this class of natural products, especially several symmetrical ring-substituted DIMs and 1,1-bis(3′-indolyl)-1- (p-substituted phenyl)methanes (methylene substituted DIMs), DIM and ring-substituted DIMs recently shown activate the aryl hydrocarbon receptor (AhR) in breast cancer cells and their activity in breast cancer may be due, in part, to inhibitory AhR–ER crosstalk (Chen et al., 1998; Safe et al., 2008; Hall et al., 2010). Thus, we synthesized and evaluated the anti-cancer activity of Phemindole, which as in yet has not been reported to have anti-cancer activity. The structure of Phemindole is shown in **Figure 1A**. To synthesize this compound, a mixture of 4-hydroxybenzaldehyde (1 mmol), 7-methyl indole (2mmol) and I_2_ (0.2mmol) were ground together in a mortar with a pestle at room temperature for several minutes. After completion of the reaction was monitored by TLC, the mixture was treated with Na_2_S_2_O_3_ to yield solid product, which was purified by column chromatography (EtOAc: hexane = 1:9) to afford the pure product (290 mg, 79%). Physical and structural characterization data obtained for Phemindole showed its purity to be higher than 95%. Phemindole was dissolved in DMSO to make a stock concentration at 20 mM for cell culture experiments. The final DMSO in the cell culture medium was 0.1% (v/v), and the same amount of DMSO was used as a negative control. The structure of Phemindole was confirmed by IR analysis that revealed 3416, 2366, 2349, 2341, 1506, 1454, 1316, 1213, 1090, 794, 773, 584 cm^-1^. The purified compound also yielded the ^1^H NMR spectra (500MHz, CDCl_3_): δ 7.75 (br s,2H), 7.21 (d, j = 8.0 Hz, 2 H), 7.17 (d, j = 8.5 Hz, 2 H), 6.95–6.89 (m, 4H), 6.70 (d, j = 8.0 Hz, 2 H), 6.61–6.59 (m, 2H), 5.78 (s, 1 H), 1.25 (s, 6 H, 2 CH_3_); ESI-MS: 389.1 [M + Na]^+^; Anal. Calcd. For C_25_H_22_N_2_O (366.45): C, 81.94; H, 6.05%; found: C, 81.80; H, 6.15%.

### Cell Culture, Reagents and Transfection

Normal Breast Epithelial cell line MCF-10A was kindly gifted by Tamara Lah and Dr. Neža Podergajs, National Institute of Biology, Ljubljana, Slovenia, MDAMB-231 cell line was obtained from the National Centre For Cell Science (NCCS), India. Cells were maintained and propagated in DMEM with various supplements as suggested by NCCS and kept at 37°C with 95% humidified air and 5% CO_2_. Culture medium was changed twice weekly and cells were maintained in complete media, until reaching 90% confluence. cDNA en-coding full-length human STIM1 (a generous gift from Dr. Paul Worley John Hopkins University) was transfected in to MDAMB-231 cells using Lipofectamine 2000 in Opti-MEM medium as per supplier’s instructions and assayed after 24 h. Isolation of stably expressing clones were done by limiting dilution and selection with G418 (500 mg/ml), and the cells that survived were cloned and assessed for STIM1 expressions by Western blot analysis. MDAMB-231 cells were transfected with 300 pmol of STIM1 siRNA (Santa Cruz Biotechnology) and Lipofectamine 2000 (Invitrogen) separately for 12 h. Levels of STIM1 proteins were estimated by Western blotting.

### Cell Viability Assays

For viability assays, cells were seeded on 96-well plates at a density of 0.5 × 10^5^ cells/well. Cell viability was measured by using the MTT cell proliferation assay kit (HIMedia). Absorbance was read at 570 nm (630 nm as a reference) on a microplate reader (Molecular Devices). Cell viability was expressed as a percentage of the control culture.

### Determination of Cell Cycle by Flow Cytometry

Briefly cells were plated at a density of 1 × 10^6^ cells/ml in each Petri dish in DMEM for 16–20 h. Thereafter the cells were treated with Phemindole at 10 μm for 24 h. At the end of treatment the cells were collected and fixed with 100% methanol for 5 min at -20°C. After centrifugation, the cell pellets were collected with 4 μg/ml of propidium iodide solution containing 100 μg/ml of RNase and 1% Triton X-100 for 30 min. Subsequently, the samples were analyzed in a FACS calibur system (BD Biosciences) using CellQuest software. The percentage of cell cycle phases was analyzed by ModFit LT software (version 2.0, BD Biosciences). Histogram display of DNA content (*x* axis, PI fluorescence) versus counts (*y* axis) has been displayed. CellQuest statistics was employed to quantitate the data at different phases of cell cycle.

### Determination of Cellular Apoptosis by AnnexinV

For the determination of cell death, cells were stained with propidium iodide and annexin V-FITC (BD Pharmingen) and analyzed on a flow cytometer (FACS Calibur, BD Bioscience) equipped with 488 nm argon laser light source, using Cell Quest Software (BD Biosciences). Electronic compensation of the instrument was done to exclude overlapping of the emission spectra. Ten thousands total events were acquired for analysis using Cell Quest software. Annexin V/FITC positive cells were regarded as apoptotic cells.

### Detection of Mitochondrial Membrane Potential and Intracellular Reactive Oxygen Species (ROS) Generation

The changes in mitochondrial membrane potential were determined using JC1 (Molecular probes). Cells were treated with DMSO or Phemindole for indicated time periods, harvested, washed twice in PBS, resuspended in PBS supplemented with JC1 (20 nM), incubated at 37°C for 15 min in the dark, and immediately analyzed by flow cytometry or fluorescence microscope. The intracellular accumulation of ROS was examined by flow cytometry after being stained with the fluorescent probe, DCFH-DA (2,7-dichlorodihydro-fluorescein di acetate; Molecular probes) (10 μM). DCFH-DA was deacetylated in cells by esterase to a non-fluorescent compound, DCFH, which remains trapped within the cell and is cleaved and oxidized by ROS in the presence of endogenous peroxidase to a highly fluorescent compound, DCF (2,7-dichlorofluorescein). Briefly, MDAMB-231 cells were seeded in 6-well plates (5 × 10^5^ cells/ml), treated with or without MPB and other compounds for different time periods, and incubated with 10 μM DCFH-DA for 30 min at 37°C. Cells were washed, re-suspended in PBS, and ROS levels were determined using FACS Calibur flowcytometry.

### Immunoprecipitation and Western Blotting

MDAMB-231 and MDAMB-468 cells were lysed in buffer (10 mm Hepes, pH 7.9, 1.5 mm MgCl_2_, 10 mm KCl, and 0.5 mm DTT) and nuclei were pelleted by a brief centrifugation. The supernatant was spun at 100,000 × *g* to get a cytosolic fraction. The nuclear extract was prepared in buffer containing 20 mm Hepes, pH 7.9, 25% (v/v) glycerol, 420 mm KCl, 1.5 mm MgCl_2_, 0.2 mm EDTA, 0.5 mm DTT, and 0.5 mm PMSF. All buffers were supplemented with protease and phosphatase inhibitor mixtures. For direct Western blot analysis, cell lysates of the particular fractions containing 30 μg of protein were separated by SDS-polyacrylamide gel electrophoresis and transferred to PVDF membrane. The protein levels were determined with specific antibodies (Santa Cruz Biotechnology). For immunoprecipitation studies STIM1 and Orai1 immune complexes were immunoprecipitated using Orai1 antibody with Protein A-Sepharose beads (Sigma). The immunopurified were immunoblotted with STIM1 antibody. The protein of interest was visualized by chemiluminescence. Equal protein loading was confirmed by reprobing the blots with β-actin/histone H1/GAPDH antibody (Santa Cruz Biotechnology).

### Calcium Measurements

Cytosolic calcium concentration was measured using a fluorimetric ratio technique. Cells were subjected to centrifugation and then re-suspended in phosphate-buffered saline (PBS) supplemented with 1 mg/ml of bovine serum albumin and incubated with Fura-2AM (final concentration 5 μM) (Sigma) in the absence of light for 30 min at room temperature. This was followed by centrifugation and cells were then re-suspended in calcium-free Hanks’ buffered saline solution (135 mm NaCl, 5.9 mm KCl, 1.2 mm MgCl_2_, 11.6 mm Hepes, 11.5 mm glucose adjusted to pH 7.3 with NaOH) prior to measurements. After centrifugation cells were again suspended in 3 ml of Hanks’ buffered saline solution in a quartz cuvette and analyzed in a Hitachi spectrofluorimeter equipped with a stirring apparatus and a thermostatted (37°C) cuvette holder, connected to a computer. The fluorescence was recorded at 510 nm using an excitation source of 340 or 380 nm. Maximum Fura-2 fluorescence (*F*_max_) was observed on addition of 1 μm ionomycin (Sigma) to the cell suspension along with 10 mm CaCl_2_, and minimum fluorescence (*F*_min_) was determined without added calcium in the presence of 5 mm EGTA (Sigma). The cytosolic [Ca^2+^] was calculated from the Fura-2AM fluorescence intensity as: [Ca^2+^]_cyt_ = *K_d_* (*F* - *F*_min_)/(*F*_max_ - *F*), where *K_d_* = 229 nm for Fura-2 and *R* is the ratio of fluorescence values (*F*) (*R* = F340/F380).

Time Lapse calcium kinetics measurement using Fluo-3AM was performed in Leica Confocal microscope. Fluo3-AM loaded cells were imaged using a Leica Confocal system attached to a Leica inverted microscope. The Fluo3-AM indicator was excited by the 506 nm. The emitted fluorescence was measured at wavelengths of ∼526 nm. Fluorescence signals were captured (1024 pixels × 1024 pixels) in real time. Time series Confocal optical sections were collected in the image-scan x-y-t mode. Planar images were recorded at specific time intervals (15 s). Background signals from auto fluorescence were adjusted to a low level by using unstained cells before image acquisitions.

### Scanning Electron Microscopic (SEM) Study

The morphology of breast carcinoma cells under different conditions were observed under scanning electron microscope (SEM). By following the protocol of Bhattacharya et al. (2015) the cells were grown on poly-L-Lysine (1:10) coated glass slide (1 cm × 1 cm) overnight at 37°C in 5% CO_2_. Next day, cells were treated with specific dose of Phemindole and kept in the CO_2_ incubator for overnight. Next day, the cells were washed by sodium cacodylate buffer, and then fixed with 2.5% glutaraldehyde buffered in sodium cacodylate for 1 h. The samples were washed with sodium cacodylate buffer thrice, and dehydrated through a series of alcohol concentrations (10, 30, 50, 70, 90, and 100%), and subjected to air drying. The samples were then visualized by EVO-18 special edition SEM (ZEISS, Germany).

### Transwell Migration Assay

Migration assay was done using cell culture inserts (BD Biosciences, Sparks, MD, USA). Cell culture inserts with pores (8 μm) were placed in the 12-well companion plate containing 100 ml of DMEM Media without chemo-attractant, i.e., serum. In the upper half of the insert 2 × 10^5^ cells was placed inside the chamber. The serum containing DMEM (10% FBS) was then added to the lower chamber of the 12-well plate. Next day the cells in the insert were washed with PBS, fixed with 3.7% formaldehyde and were permeabilized using methanol. At last, cells were stained with Giemsa stain for 30 min. Cells present in the lower part of the inserts were determined by counting cells in three microscopic fields per well, and the extent of migration will be expressed as an average number of cells per microscopic field.

### Wound Healing Assay

The migration of breast cancer cells under different conditions were determined by bidirectional wound healing assay. Firstly the MDAMB-231 cells were grown to 90% confluency in 12 well plates after which a scratch was done using sterile pipette tip to form a bidirectional wound. Migration was quantitated by a semi-automated, computer-assisted procedure by a person blinded with respect to the experimental treatment. The data from triplicate wells were calculated as the means ± SEM, the migration rate of control cells were taken as 100% and healing rate of other plates were compared with respect to control cells.

### Fluorescence Imaging

For fluorescence imaging, the cells were fixed with 3.7% formaldehyde and permeabilized with 0.1% Triton X-100. Cells were then either incubated with anti STIM1, ORAI1 antibody, anti cytochrome C, p-FAK antibody followed by FITC/TRITC-conjugated secondary antibody (Sigma). The nucleus was stained with DAPI (SRL). The samples were mounted on clean glass slides using Vector Shield mounting Media and visualized under the fluorescence microscopes (Leica). Images were quantified using this formula to calculate the corrected total cell fluorescence (CTCF). CTCF = Integrated Density – (Area of selected cell X Mean fluorescence of background readings).

### Ethics Statement

The study was performed under strict accordance with the protocols of the National Institute of Health guidelines for the Care and Use of Laboratory Animals (NIH publication No. 85 -23 revised 1985: US Department of Health, Education and Welfare, Bethesda, MD, USA). The experimental outline also met the National Guidelines on the Proper Care and Use of Animals in Laboratory Research (Indian Science Academy, New Delhi, India) and the protocol was approved by the Institutional Animal Ethics Committee (IAEC) of Bose Institute, Kolkata, India (Approval No. IAEC/BI/08/2012). The animal breeding and experimental facility are registered with the Committee for the Purpose of Control and Supervision of Experiments on Animals (CPCSEA), Ministry of Environment and Forest and Climate Change, Government of India. Euthanasia was performed by decapitation under sodium pentobarbital anesthesia, and the efforts were made to minimize the pain and sufferings to the animals.

### Tumor Regression in *Ex Ovo* Model

The semi-quantitative Chorioallantoic Membrane (CAM) assay was performed as described (Zijlstra et al., 2002) with a few modifications to detect the presence of human cancer cells. Briefly, 2 × 10^6^ MDAMB-231 cells were inoculated on the CAM of 6-day old chicken embryos. A tumor was observed within 2 days. On day 10 and 12, chicken embryos were treated intravenously with Phemindole at a dosage of 10 μm/ml. Untreated embryos served as control. On day 15, the embryos were sacrificed. To detect human cells in the chick tissues, primers specific for the human alu sequences were used to amplify the human *alu* repeats present in genomic DNA against chick GAPDH as loading control.

### 4T1 Induced Tumor Regression Study in BALB/C Model

Sixteen female BALB/c mice (8 weeks old and weighing ∼20 g) purchased from Chittaranjan National cancer research institute and housed in the animal house of Bose Institute and were maintained according to the guidelines of the Institutional Animal Ethical Committee (CPCSEA). After acclimatization under the laboratory condition, they were randomly divided into four groups, each group having four mice (N ¼ 4) (i) Untreated control group. (ii) untreated 4T1tumor bearing set (which were injected with 4T1 cells in mammary fat pads), (iii) 10 mg/Kg bodyweight Phemindole treated tumor bearing set (iv) 15 mg/Kg bodyweight Phemindole treated tumor bearing set. The Phemindole dissolved or suspended in 0.1 mL PBS were injected subcutaneously on the inside of either mammary fat pads every 48 h with a 25 gage syringe. Each mouse received 7 doses of its designated drug over a 14 days period. Mice in the untreated group were similarly given 7 doses of 0.1 mL of PBS. Tumor volume was measured on every date of treatment and tumor weight was evaluated following sacrifice of the mice. Blood was collected from the retro-orbital plexus of anesthetized mice of each group by rupturing it with a fine capillary and was kept at 4°C for 1 h in slant position. It was centrifuged at 2000 rpm for 10 min at 4°C. Clear serum formed above the clot was collected. Serum specific Liver and Kidney toxicity markers were measured using commercially available standard kits purchased from Himedia, India following manufacturer’s protocol. For histological studies, the liver, kidney, spleen, tissues collected from all the groups were fixed with 10% phosphate buffered neutral formalin. Following dehydration in graded (50–100%) alcohol, the tissues were embedded in paraffin. Thin tissues sections, having 4–5 μM thickness, were cut and stained with routine haematoxylin and eosin (H&E) stain for photo microscopic assessment.

### Statistics

Values are shown as SE except where otherwise indicated. Data were analyzed, and when appropriate, significance of the differences between mean values was determined by Student’s *t*-test, one way ANOVA, Results were considered significant at *p* < 0.05.

## Results

### Phemindole Reduced Cell Viability and Induced Cell Apoptosis in Human Triple Negative Breast Cancer Cells

In order to assess the effect of Phemindole (**Figure 1A**) on TNBC cells MTT assay was performed. Interestingly our observations revealed significant death of the MDAMB-231 with increasing concentrations of Phemindole and the percentage of cell death was substantially higher at even lower doses in comparison to that of 3,3′di Indolyl methane (DIM) (**Figure 1B**). Results of this figure clearly revealed that Phemindole induced nearly 50% cell death as compared to only 20% with DIM at a dose of 10 μM on treatment for 24 h. In contrast the percentage of normal breast epithelial MCF-10A cell survival as assessed by MTT assay was significantly higher when subjected to Phemindole treatment (**Figure 1B**). These findings thereby established the fact that the synthesized indole derivative Phemindole showed prominent cytotoxicity in TNBC cells at a dose in which it left the normal breast epithelial cells (MCF-10A) unaffected. Additionally, our findings also highlighted that Phemindole creates potent toxicity in TNBC cells (MDAMB-231) more efficiently at a much lower doses than DIM alone. To further reinstate the findings obtained from MTT assay, we performed phase contrast microscopic imaging of MDAMB-231 cells on treatment with 10 μM dose of Phemindole. Interestingly our observations revealed that Phemindole produced significant level of morphological change to induce death in TNBC cells (**Figure 1C**). It has been acknowledged that in addition to loss of membrane integrity, apoptotic cells can also be distinguished based on morphological hallmark such as membrane blebbing and apoptotic body formation (Azimi et al., 2014). Thus we also monitored the changes in cellular morphology, using SEM and our observations revealed the formation of apoptotic body and membrane blebbing, a prominent indication of cell apoptosis which is quite evident from the SEM micrograph as shown in **Figure 1D**. To further determine whether the increase in percentage of cell death was due to apoptosis or not we used flowcytometry to analyze the number of Annexin-V-positive cells. Results illustrated in **Figure 1E** showed a significant amount of increase in the number of Annexin-V-positive MDAMB-231 cells, indicating apoptosis as the primary cause of Phemindole mediated cell death. These result thereby indicated that Phemindole significantly induced apoptosis in MDAMB-231 cells at a dose 10 μM.

### Phemindole Induced Apoptosis via ROS Generation through Mitochondria Mediated Pathway

**FIGURE 2:**
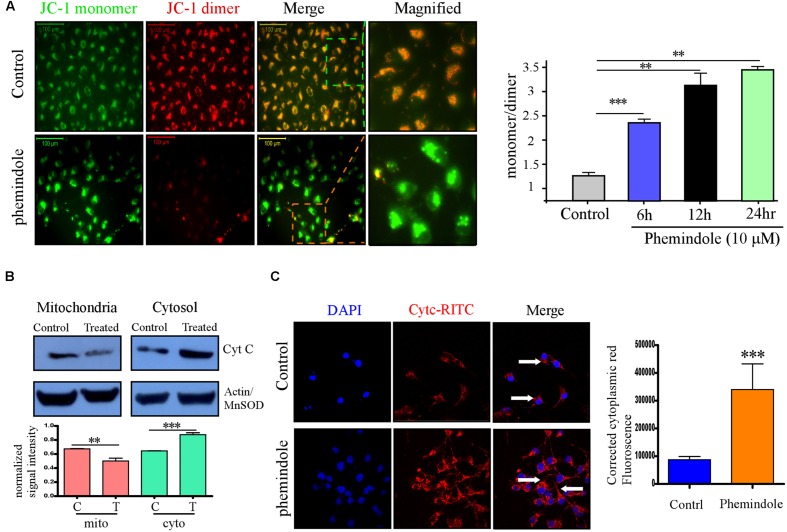
**Phemindole potentiates mitochondrial membrane potential depolarization and Cytochrome C release in MDAMB-231 cells.** **(A)** Fluorescence microscopic images of the MDAMB-231 cells stained with JC-1 dye for detecting the changes in mitochondrial membrane potential in both control and Phemindole treated cells. The red fluorescence intensity in the Phemindole treated cells was weaker than in the control cells (Left panel). Bar diagram shows the quantitative monomer (Red Fluoroscence)/dimer (green fluorescence) ratio at different time point of Phemindole (10 μM) treated set (Right panel). **(B)** Western blot assay showing Cyt C release (upper panel). The level of Cyt C increased in the cytoplasm and decreased in the mitochondria of the MDAMB-231 cells treated with the Phemindole, indicating that Phemindole caused the release of Cyt C from mitochondria. β-actin for cytosol and Mn-SOD for mitochondria were taken as positive control. Blots are representative of three independent experiments and quantifications are indicated by Bar diagram (lower panel). Values were normalized by intensity of positive control. **(C)** Immunofluorescence images of Cyt C –TRITC (Red) and DAPI (blue) staining in MDAMB-231 cells from untreated and Phemindole treated sets (Left panel). Pictures showed representative cells of each population and were representative of three independent experiments. Corrected cytoplasmic red Fluorescence intensity was quantified following the protocol described in material method section (Right panel). ^∗^, ^∗∗^, ^∗∗∗^ indicate *p* < 0.05, *p* < 0.01, *p* < 0.0005 versus untreated cells.

**FIGURE 3:**
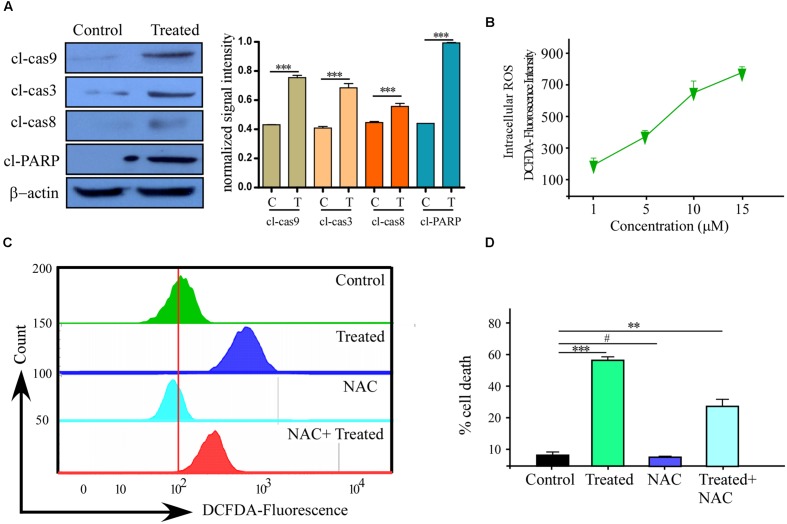
**Reactive oxygen species (ROS) production with Caspase activation is one of the reason of Phemindole mediated MDAMB-231 cells apoptosis.** **(A)** Western blot analysis of cleaved caspase-3, cleaved caspase-9, cleaved caspase-8 and cleaved-PARP in control and Phemindole treated MDAMB-231 cells (Left panel). β-actin was used as loading control. Blots are representative of three independent experiments and quantifications are indicated by Bar diagram (Right panel). Values were normalized by intensity of positive control. **(B)** Line diagram representation of the dose dependent ROS production in MDAMB-231 cells on treatment with Phemindole. **(C)** Phemindole treated MDAMB-231 cells were assessed by flow cytometry for ROS generation by measuring the DCF-fluorescence intensity and the data was represented by histogram overlay. **(D)** Percentage of cell death from Phemindole induced with/without NAC (a pharmacological inhibitor of ROS) was assessed and represented in bar diagram. As described in methods the statistic data were presented as mean ± SE from three independent experiments. ^∗^,^∗∗^,^∗∗∗^,^#^ indicate *p* < 0.05, *p* < 0.01, *p* < 0.0005, *p* < 0.05 respectively versus untreated or control group.

To further explore the anti-tumor activity of the Phemindole, we were interested in studying the detailed molecular mechanism of induction of apoptosis in TNBC cells. Immuno-fluorescence images of cells stained with JC-1 indicated a drop in the mitochondrial membrane potential more significantly in MDAMB-231 cells treated with Phemindole (**Figure 2A**, left panel). **Figure 2A** (right panel) summarizes our finding that the fluorescence intensity ratio of red to green declined dramatically in Phemindole treated cells clearly indicating a loss in the mitochondrial membrane potential of the TNBC cells. Additionally, we checked for Cyt C expression which is deemed to be one of the most significant factors in the mitochondria mediated apoptosis cascade. Western blotting analysis revealed an increased expression of Cyt C in the cytosol (**Figure 2B**) which was further validated from our Immunofluorescence imaging data. In untreated cells, Cytochrome c-FITC had a very weak plaque like signal, while an intense signal was evident in the cytoplasm of the Phemindole treated MDAMB-231 cells (**Figure 2C**). This undoubtedly proved the fact that Phemindole treatment facilitated the cytosolic diffusion of Cyt C from the mitochondria which subsequently initiated the cleavage of Caspase 8, Caspase-9, Caspase-3 and finally PARP cleavage (**Figure 3A**) to induce apoptosis in the MDAMB-231 cells. It is well established and also supported by many scientific reports that high levels of ROS formation usually initiate the mitochondrial membrane potential disruption of many types of cells (Stowe and Camara, 2009). In the present experimental set up, we first determined whether treatment Phemindole could up-regulate the formation of ROS in MDAMB-231 cells. Cells were treated with different concentrations (1, 5, 10, and 15 μM) of Phemindole, and the ROS generation was measured using the fluorescent dye DCFH-DA and the results were analyzed using flow cytometry. Treatment with 5 μM of Phemindole induced significant high levels of ROS production in MDAMB-231 cells (**Figure 3B**). To further analyze the situation and to justify the role of ROS in the apoptotic cascade, the MDAMB-231 cells were pre-treated with 5 mM concentration of NAC (*N*-acetyl Cysteine), a very potent and widely accepted antioxidant. Interestingly, our findings highlighted that treatment with NAC resulted in a sustained decrease in ROS generation which is quite close to normal. Moreover, when these NAC pre-treated cells were subjected to treatment with Phemindole, their ROS generation was prominently declined in comparison to Phemindole treatment alone (**Figure 3C**). Therefore, from the above observation it may be concluded that Phemindole failed to initiate ROS generation in NAC pre-treated cells and thus the percentage of apoptotic cell death was also subsequently declined (**Figure 3D**). Interestingly, results of **Figure 3D** clearly revealed that NAC pretreated followed by Phemindole treated cells showed reduction in percentage of cell apoptosis to nearly 25%, whereas only Phemindole treatment alone showed about 60% of cell apoptosis thereby suggesting the probable involvement of any other signaling cascade in addition to the mitochondria mediated ROS generation pathway. Thus we were further prompted to extend our studies to delineate the involvement of other cellular pathway which may be coupled with the ROS generation pathway by Phemindole to induce substantial apoptosis in TNBC cells.

### STIM1 Was Down Regulated in Phemindole Treated MDAMB-231 Cells

**FIGURE 4:**
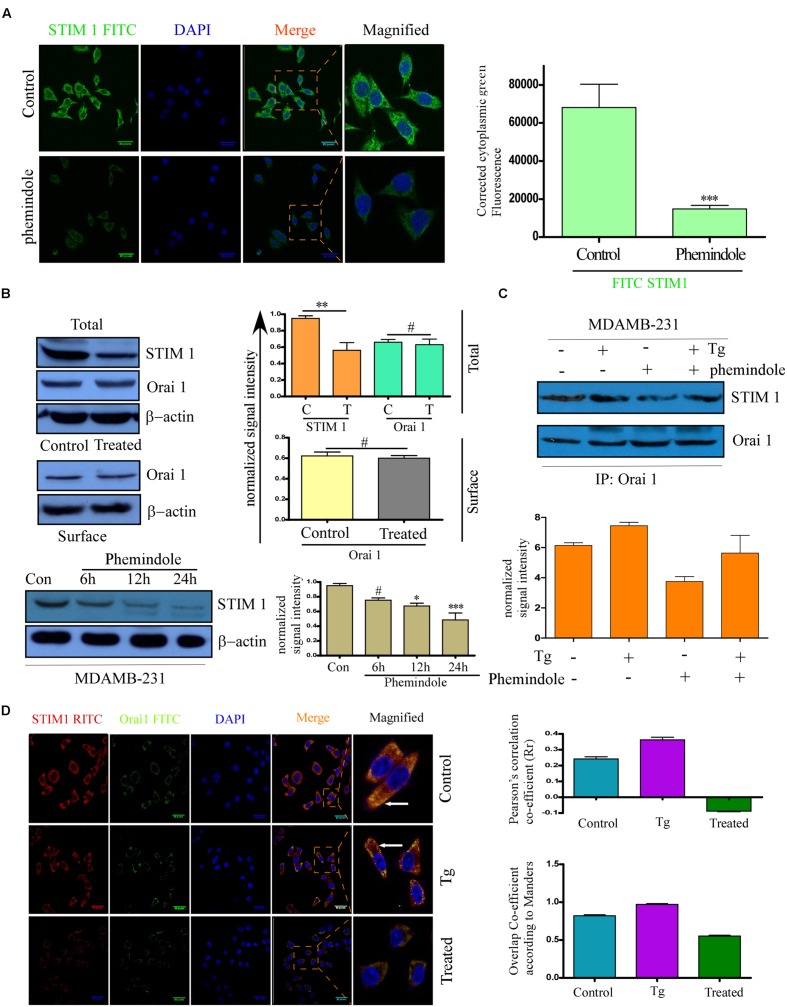
**Phemindole downregulates STIM1 Expression.** **(A)** Immunofluorescence images showing decreased expression of STIM1 in the MDAMB-231 cells in response to Phemindole treatment. Anti STIM1 antibody was FITC tagged (green) and nuclei were stained with DAPI (blue) (left panel). Corrected Fluoroscence intensity of STIM1 protein was quantified and represented as bar diagram (Right panel). Pictures showed representative cells of each population and were representative of three independent experiments. **(B)** Western blot analysis depicting the changes in the expression pattern of STIM1 and Orai1. MDAMB-231 cells were treated with Phemindole and Western blotted with respective antibodies as labeled in the figure (upper-left panel). Quantification of WB were depicted as bar diagram (upper-right panel). MDAMB-231 cells were treated with 10 μM Phemindole for 24 h. Lysates were subjected to SDS–PAGE followed by Western blotting (with anti-Orai1 antibodies. β–actin was used as a loading control (middle-left panel). Band intensity was quantified and represented as a bar histogram (middle-right panel). Cells were treated with 10 μM Phemindole and were harvested at 0, 6, 12, or 24 h after Phemindole treatment. Western blotting was done. Antibodies used are labeled and β-actin was used as loading control (lower-left panel). Lower-right panel represent the normalized intensity of the bands. Blots are representative of three independent experiments and quantifications are indicated by Bar diagram. Values were normalized by intensity of loading control. **(C)** MDAMB-231 cells were treated with Phemindole or Tg under different conditions, lysed and proteins were subjected to immune precipitation by anti STIM1 antibody and followed by Western blotting with respective antibody (upper panel). **(D)** Representative Fluorescence images showed the association between STIM1 (red) and ORAI1 (green) at plasma membrane. Nuclei were stained with DAPI (blue). Enlargements of the areas indicated by the color rectangles showed overlap formation of STIM1 and Orai1 in plasma membrane due to Tg treatment (Left-middle) which is invisible in Phemindole treated set (left-lower) and not prominent in un-stimulated control set (left-upper panel). Pictures showed representative cells of each population and were representative of three independent experiments. Right panel, the Pearson’s and Manders coefficients determining the level of overlap of RITC and FITC on treatment with Control, thapsigargin (*TG*) and Phemindole has been represented graphically. ^∗^,^∗∗^,^∗∗∗^,^#^ indicate *p* < 0.05, *p* < 0.01, *p* < 0.0005, *p* > 0.05 respectively versus untreated or control group.)

After elucidating the role of Phemindole in inducing cell death via ROS generation in a mitochondrial-dependent pathway, we again raised the question about the involvement of some other pathway which may be coupled with the ROS generation in inducing substantial death in MDAMB-231 cells. It is well known that store-operated Ca^2+^ entry (SOCE) is crucial for maintaining intra cellular calcium homeostasis and functions (Selvaraj et al., 2015). Stromal interaction molecule 1 (STIM1), acts as a key component of SOCE, by controlling a dual mechanism as an ER Ca^2+^ receptor and an SOCE exciter. Differential expression of STIM1 has been discovered in several human cancer cells (Azimi et al., 2014; Sun et al., 2015; Wen et al., 2016). Keeping this in mind, we were interested to evaluate the role of Phemindole on SOCE in MDAMB-231 cells. To investigate whether Phemindole has any effect on the expression of STIM1 in MDAMB-231 cells, we performed the immuno-fluorescence staining and western blot of STIM1. On immuno-staining (IF), we observed that, MDAMB-231 cells showed a reduction in fluorescence of STIM1 on treatment with Phemindole (**Figure 4A**). Western blotting results also reinstated our findings obtained from our immuno-fluorescence data (**Figure 4B**, upper panel). Simultaneously we also checked the expression of Orai1 in control and Phemindole-treated MDAMB-231 cells. Importantly, Phemindole treatment produced no alteration in the total or surface expression of Orai1 (**Figure 4B**, middle panel). However, a decrease in total STIM1 levels in a time-dependent manner was observed in the Phemindole treated TNBC cells (**Figure 4B**, lower panel). Previous reports suggest that upon store-depletion, STIM1 is transported to the ER–PM junctions where it coordinates with Orai1 channels (Carrasco and Meyer, 2011). Thus, co-immunoprecipitation experiments using STIM1 antibodies were carried out under control and store-depleted conditions. Addition of the commercially available SERCA pump inhibitor, thapsigargin (Tg), resulted in an enhancement in STIM1–Orai1 interaction in MDAMB-231 cells, which was declined on Phemindole treatment (**Figure 4C**). These data henceforth suggested that Phemindole could inhibit STIM1–Orai1 functional interaction that resulted in inhibition of Ca^2+^ entry and induced cell death in TNBC cells. The findings was also re-evaluated by double immuno-staining of MDAMB-231 cells in following three condition untreated, Tg treated, Phemindole treated (**Figure 4D**).

### Store Operated Calcium Entry Was Also Suppressed in Phemindole Treated MDAMB-231 Cells

**FIGURE 5:**
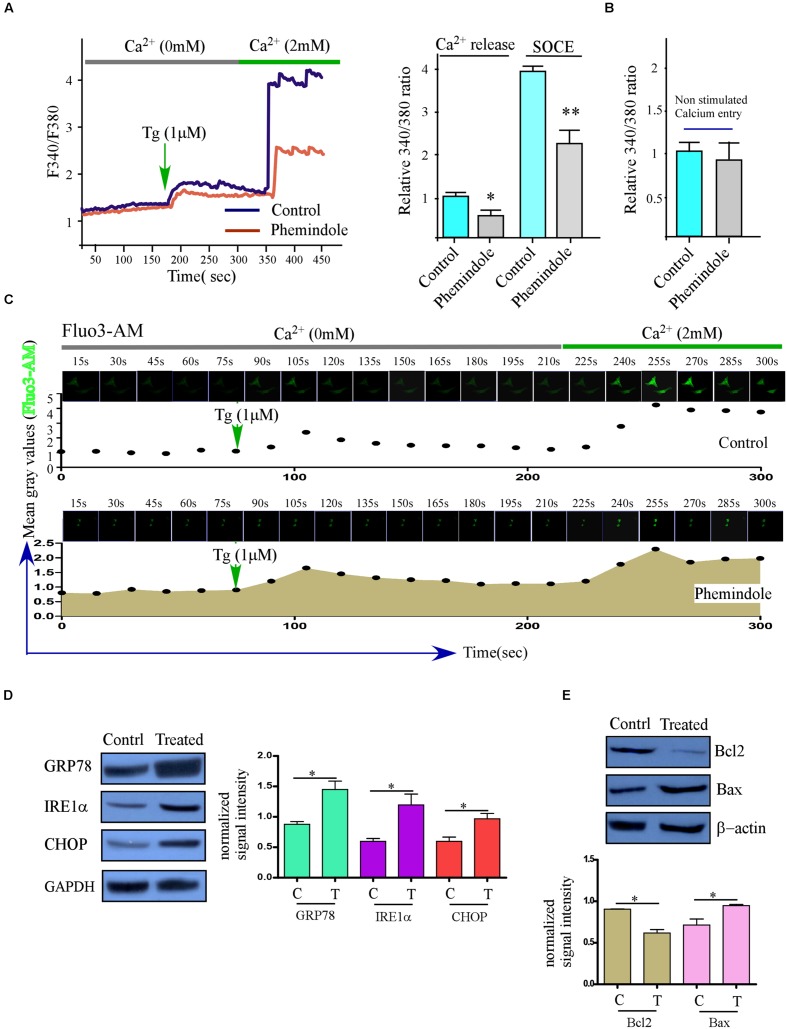
**The treatment of TNBC cancer cells with Phemindole reduces SOCE and induces ER stress.** **(A)** Ca^2+^ measurement was performed in control and in the presence of Phemindole (10 μM) in MDAMB-231 cells. Analog plots of the fluorescence ratio (340/380) from an average of 40–60K cells are shown (Left panel), Bar diagram showed the qualitative values of fluorescence ratio (340/380) during calcium efflux and SOCE after TG treatment (Right panel). The statistic data were presented as mean ± SE from three independent experiments. ^∗^*p* < 0.05, versus control group. **(B)** Non-stimulated Ca^2+^ influx of control and Phemindole treated sets were assessed and fluorescence ratio were presented as a bar diagram. Data shown as average ± SE (*n* = 3). **(C)** Time Lapse calcium kinetics images depicting the changes in calcium influx in control and Phemindole treated MDAMB-231 cells. **(D)** Western blot was performed to show the expression of unfolded protein response (UPR) related protein (GRP78, IRE1α, and CHOP) in the TNBC cancer cells after 10 μM Phemindole treatment for 24 h. **(E)** Western blot analysis determining the changes in Bax and Bcl-2 expression levels in MDAMB-231 cells. β-actin was used as internal loading control. Blots are representative of three independent experiments and quantifications are indicated by Bar diagram (lower panel). Values were normalized by intensity of loading control. ^∗^,^∗∗^,^∗∗∗^,^#^ indicate *p* < 0.05, *p* < 0.01, *p* < 0.0005, *p* > 0.05 respectively versus untreated or control group.

Stromal interaction molecule1 activated Orai1 by interacting with it at plasma membrane, and this interaction was crucial for SOC-mediated Ca^2+^ entry (Selvaraj et al., 2015). Keeping this thought in mind we next investigated whether SOCE is altered upon Phemindole treatment as Phemindole was already found to down regulate STIM1 expression. To evaluate SOC-mediated Ca^2+^ entry, we treated the cells with thapsigargin (Tg, 1 μM) to bring about depletion of the ER Ca^2+^ stores. Our observation revealed that in the absence of extracellular Ca^2+^ the increase in [Ca^2+^]i evoked by Tg (first peak) was prominently declined on Phemindole treatment (**Figure 5A**, Left panel). Simultaneously, even upon addition of external Ca^2+^(1 mM), which initiated the SOC-mediated Ca^2+^ entry, the [Ca^2+^]i was also declined (second peak) in Phemindole treated MDAMB-231 cells (**Figure 5A**, right panel). To address the question that whether Phemindole treatment can also alter the constitutive calcium influx in MDAMB-231 cells we repeated the previous experimental condition except the thapsigargin stimulation. **Figure 5B** showed that there was no significant difference in calcium influx ratio between normal and Phemindole treated MDAMB-231 cells. Additionally our Fluorimetric data was revalidated using time Lapse calcium kinetics measurements and changes in the calcium influx in control and Phemindole treated sets have been illustrated in **Figure 5C**. These results indicated that SOCE was inhibited on treatment with Phemindole, which could be an important factor to restrict cell proliferation.

### Phemindole Treatment Induced ER Stress

Our previous results have shown that Phemindole disrupted cellular calcium homeostasis by diminishing SOC mediated ER calcium entry in the cells. These findings further encouraged us to investigate whether this loss in ER–Ca^2^ provoked the unfolded protein response (UPR) (Bravo et al., 2013; Selvaraj et al., 2015). Our findings interestingly revealed that the UPR markers (GRP78 and CHOP) were elevated significantly at the protein levels (**Figure 5D**) on Phemindole treatment. Because ER stress mediated CHOP activation has been known to be one of the main causes of pro apoptotic protein Bcl2 down regulation (Tabas and Ron, 2011), we next evaluated the changes of expression of these proteins by western blot and immunostaining and as shown in **Figure 5E** Phemindole treatment altered Bax, Bcl2 ratio by decreasing the expression of Bcl2.

### Decreased Ca^2+^ Entry in MDAMB-231 Cells Induced Cell Death and Restoration of STIM1 Expression Reverted Back the Phemindole-Mediated Restriction of Cell Proliferation and Survival of TNBC Cells

**FIGURE 6:**
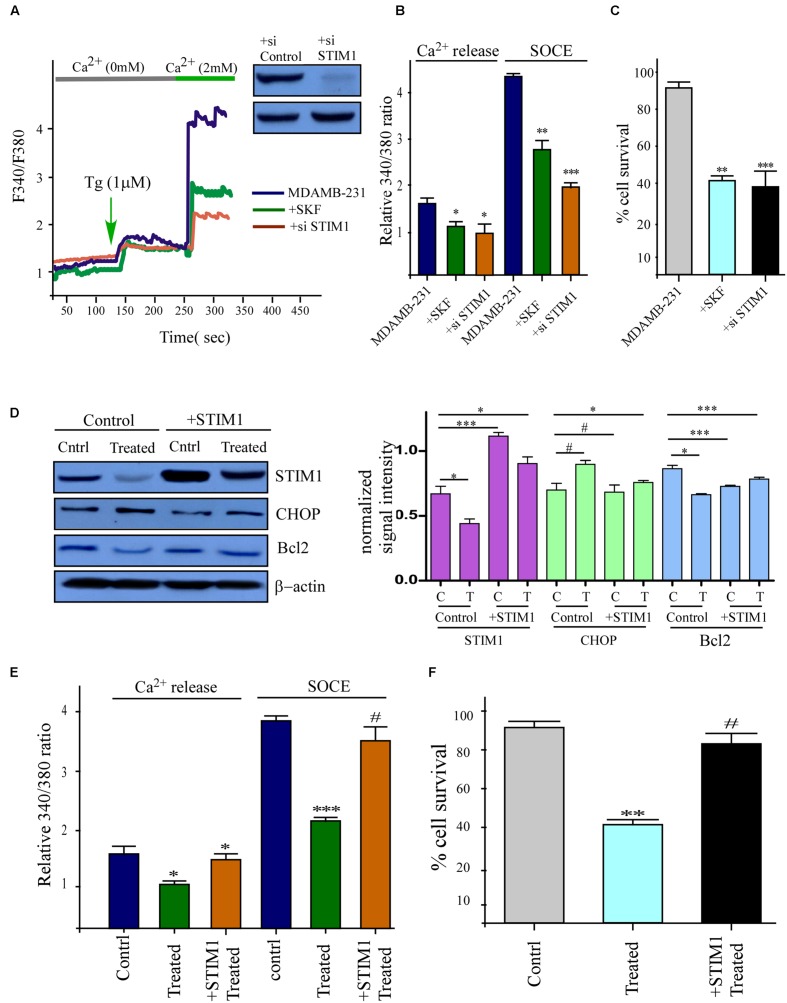
**Inhibitions of STIM1 mimics the effects of Phemindole and over expression of STIM1 showed opposite effect.** **(A)** Ca^2+^ imaging was performed in control, in the presence of SKF 96365 (SOCE inhibitor) and STIM1 si-RNA treated MDAMB-231 cells. Analog plots of the fluorescence ratio (340/380) of the cells were shown. Knockdown of STIM1 protein was confirmed by western blot analysis (Inset) **(B)** Quantification (mean ± SD) of fluorescence ratio (340/380). ^∗^*P* < 0.05 versus control. **(C)** Bar diagram showed MTT assay under SKF 96365, STIM1 si-RNA, treated conditions for 24 h in MDAMB231 cells. **(D)** STIM1 protein levels were restored by Transfection of STIM1 clones and also other protein levels were measured in presence and absence of Phemindole in STIM1 transfected cells by western blot (left panel). Quantification of band intensity in respect to loading control was shown in bar diagram (left panel). Blots are representative of three independent experiments and quantifications are indicated by bar diagram. Values were normalized by intensity of loading control. **(E)** Fura-2 Ca^2+^ imaging showing the results of a quantitative analysis of Ca^2+^ content (or Ca^2+^ release in after Tg treatment) and Ca^2+^ influx during SOCE as mentioned previously. Values are the mean ± SEM for all cells in plates ^∗^*p* < 0.05 versus control group. **(F)** Bar diagram represented the cell proliferation pattern of STIM1 over expressed MDAMB-231 cells in presence of Phemindole treatment. Each bar gives the mean ± SEM of three separate experiments. ^∗^,^∗∗^,^∗∗∗^,^#^ indicate *p* < 0.05, *p* < 0.01, *p* < 0.0005, *p* > 0.05 respectively versus untreated or control group.

To clearly interpret the link between Ca^2+^ influx and cell survival, we next aimed to inhibit SOCE. Thus we added SKF96365 in order to block SOC mediated Ca^2+^ entry (Chen et al., 2013), and this resulted in significant decline in Tg induced Ca^2+^ entry in MDAMB-231 cells. Also inhibition of SOCE by using STIM1 siRNA gave the same result as in the previous case (**Figures 6A,B**). Similarly, as expected cell proliferation was also declined under both these above-mentioned condition (**Figure 6C**). To further confirm the role of Phemindole in mediating loss in Ca^2+^ influx (due to loss of STIM1 expression) for death in TNBC cells, we over-expressed STIM1 protein in MDAMB-231 cells. Over expressed MDAMB-231 cells showed enhanced expression of STIM1 on treatment with Phemindole in comparison to the untreated cells (**Figure 6D**). However, no rise in control β-actin levels was observed. Importantly, Phemindole-dependent inhibition of SOCE was also reverted back in cells with STIM1 over-expression. Simultaneously, Bcl2 expression were also restored in these treated cells (**Figures 6D,E**). In fact, both cell survival and cell proliferation were reinstated in the cells that over expressed STIM1 with Phemindole treatment (**Figure 6F**). Cumulatively, these results suggested that elevation in STIM1 expression restored Phemindole-dependent loss in Ca^2+^ entry and inhibited Phemindole-mediated cell death in TNBC cells.

### *In Vitro* Effect of Phemindole on Cell Cycle Distribution and Retardation of TNBC Cell Migration

**FIGURE 7:**
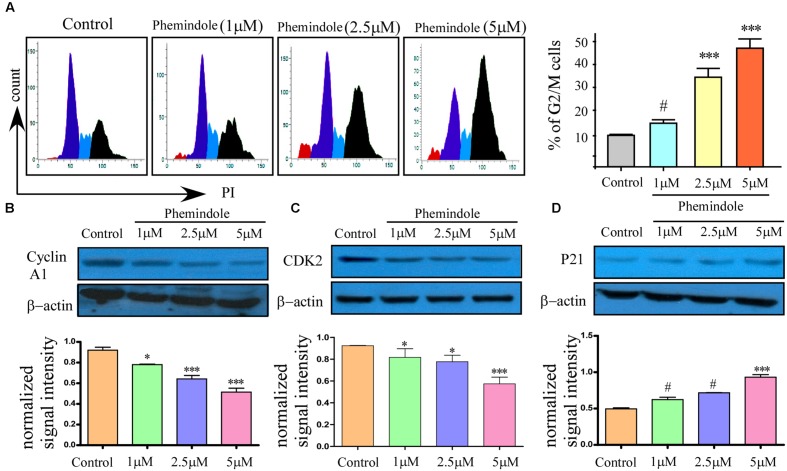
**Anti-mitotic property of Phemindole on MDAMB-231 cells.** **(A)** Phemindole induced cell cycle arrest in the G2/M phase in MDA-MB cells. Cells were treated for 24 h with Phemindole at different concentrations as indicated, cells were processed for cell cycle analysis (left panel). Quantified Graphical plot on the right panel. **(B–D)** Western blot analysis was performed to check the expression of cell cycle-associated proteins in presence of Phemindole in different concentration; β-actin was used as a loading control. Quantification of band intensity in respect to loading control was shown in bar diagram (lower panels). Blots are representative of three independent experiments and quantifications are indicated by bar diagram. Values were normalized by intensity of loading control. ^∗^,^∗∗^,^∗∗∗^,^#^ indicate *p* < 0.05, *p* < 0.01, *p* < 0.0005, *p* > 0.05 respectively versus untreated or control group.

**FIGURE 8:**
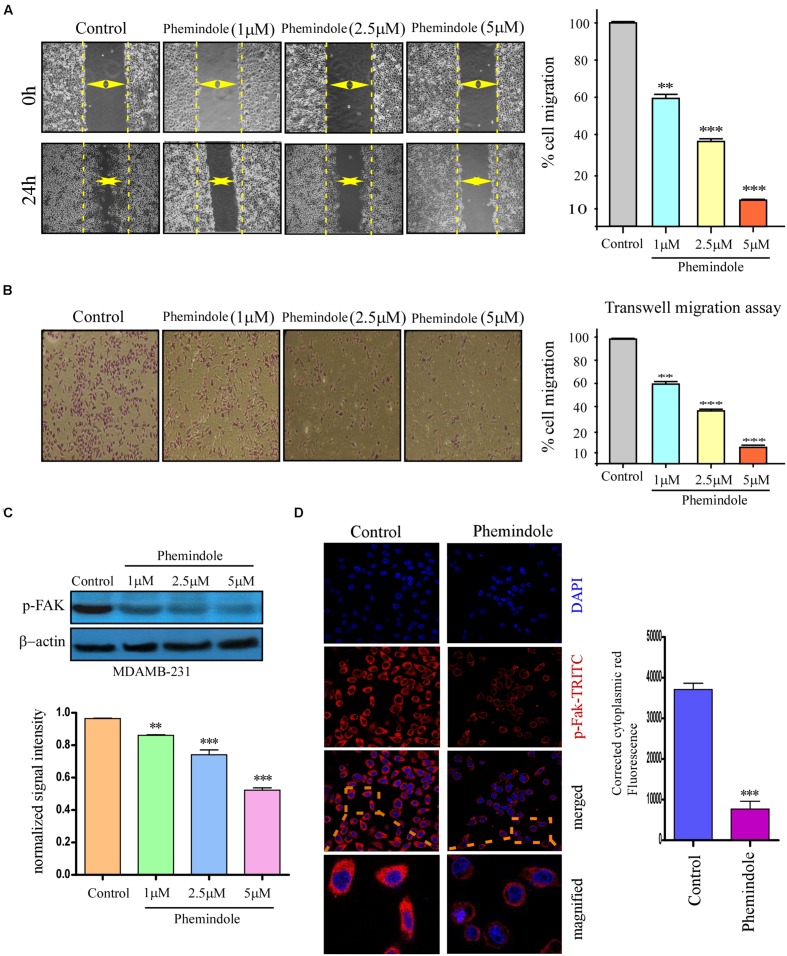
**Phemindole also shows anti migratory property on TNBC cells.** **(A)** The rate of migration of MDAMB-231 breast cancer cells, on treatment with different concentration of Phemindole (1, 2.5, 5 μM) at 0 and 24 h as determined from bidirectional wound healing assay (left panel). Bar diagram representation of the percentage of migration of MDAMB- 231 cells at 0 and 24 h on treatment with different concentration of Phemindole (1, 2.5, 5 μM) (right panel). **(B)** Phase contrast images showing the stained MDAMB-231cells as obtained from the *trans* well migration assay in control and Phemindole treated cells at different concentrations of Phemindole (1, 2.5, 5 μM) (left panel). Percentage of cell migration of MDAMB-231 cells was represented by bar chart under the aforementioned conditions (right panel). **(C)** Western blot analysis determining the changes in pFAK expression levels of the aforementioned set (upper panel). Quantification of band intensity in respect to loading control was shown in bar diagram (lower panels). Blots are representative of three independent experiments and quantifications are indicated by bar diagram. Values were normalized by intensity of loading control. **(D)** Immunofluorescence images showing decreased expression of p-FAK in the cytosolic region in response to Phemindole treatment in MDAMB-231 cells. Anti-p-FAK secondary antibody was FITC tagged (green) and nuclei were stained with DAPI (blue) (left panel). Corrected Fluoroscence intensity of p-FAK protein was quantified and represented as bar diagram (right panel). Pictures showed representative cells of each population and were representative of three independent experiments. Values are mean ± SEM of three independent experiments in each case or representative of typical experiment. ^∗^,^∗∗^,^∗∗∗^,^#^ indicate *p* < 0.05, *p* < 0.01, *p* < 0.0005, *p* > 0.05 respectively versus untreated or control group.

It is well known that MDAMB-231 cells are highly metastatic in nature (Tu et al., 2011), as shown above molecular mechanism through which Phemindole induced apoptosis in these cells, we were further motivated to study whether Phemindole have any anti mitogenic property or not. To determine the anti-mitogenic effect of Phemindole, cell-cycle analysis was performed in MDAMB-231 cells. Cells were accumulated in the G2/M phase by Phemindole in a dose-dependent manner as shown in **Figure 7A**. Five μM Phemindole produced the most eminent effect on cell cycle arrest and about 50% cells were stalled in G2/M phase. We further examined the effects of Phemindole on cell cycle arrest-related proteins by immunoblot analysis Cyclin A1, CDK inhibitor p21 are the main key players in the G2/M checkpoint (Barboule et al., 1999; Li G. et al., 2013). **Figure 7B** revealed that Phemindole treatment declined the protein level of, Cyclin A1, CDK2 (**Figure 7C**) and increased the level of p21 (**Figure 7D**) in a dose-dependent manner thereby highlighting the anti-mitogenic effect of Phemindole on TNBC cells. With the objective of investigating the anti-migratory property of Phemindole on breast carcinoma cells, we first elucidated the different doses of Phemindole in inhibiting breast cancer cell migration using *in vitro* bidirectional wound healing assay. As illustrated in **Figure 8A**, Phemindole significantly (*p* < 0.001) inhibited migration of MDAMB-231 cells even at a minimal dose of 5 μM. These findings therefore highlighted the fact that the synthesized Phemindole could prevent the *in vitro* motility of breast cancer cells at significantly lower dose of 5 μM, which is much lower than the effective apoptotic dose, i.e., 10 μM. To further confirm our above-mentioned findings the transwell migration assay was performed using MDAMB-231 cells. The cell line was treated with different doses of Phemindole (1, 2.5, 5 μM) in a separate set. Interestingly, as illustrated in **Figure 8B**, lesser number of cells were found to have been migrated to the under surface of the transwell insert when treated with 2.5 and 5 μM Phemindole in comparison to a significant high number of migratory cells in the case of control and 1 μM treated sets. Thus, we were impelled to delineate specifically the effect of Phemindole on retardation of *in vitro* breast cancer cell migration. Further to determine the molecular mechanism playing behind this finding, the status of phosphorylated Focal adhesion kinase (FAK) in the treated or untreated cells was examined. FAK is a 125-kDa non-receptor protein tyrosine kinase identified in early 1990s as a protein associated with focal adhesions (Luo and Guan, 2010). In most of the breast cancers, the elevated levels of FAK (Focal Adhesion Kinase) expression were already reported (Mitra et al., 2005). Subsequent analysis using western blot and immune-cytochemistry showed that pFAK expression has been decreased significantly in Phemindole treated cells as compared to control cells (**Figures 8C,D**). These observations discussed so far clearly reinstate our findings that Phemindole display a potent anti mitotic and anti-migratory property along with its significant anti-apoptotic nature in TNBC cells.

### *In Vivo* Validation of Tumor Suppressive Effect of Phemindole in BALB/c Model

**FIGURE 9:**
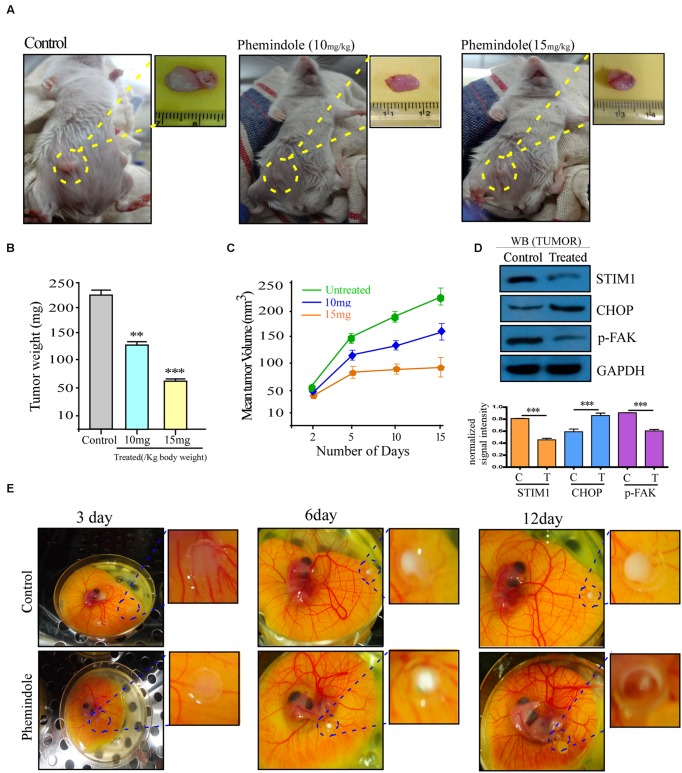
**Validation of tumor suppressive effect of Phemindole in *ex ovo* model and *in vivo* breast tumor in BALB/c mice model.** **(A)** Photograph of 4T1 cell induced tumors from BALB/c mice and excised tumors from untreated or treated with Phemindole 10 mg/kg and 15 mg/kg body weight. **(B)** Weight of the excised tumors from control and Phemindole treated sets were measured, analyzed and represented in the form of bar graph. **(C)** Graphical representation of the changes in the tumor volume of Phemindole treated and untreated mice for a period of 8 weeks. **(D)** Western blot analysis depicting the changes in the expression level of different protein level in the untreated and Phemindole treated tumors. β-actin was used as internal loading control (upper panel). Blots are representative of three independent experiments and quantifications are indicated by Bar diagram (lower panel). Values were normalized by intensity of loading control. **(E)** *Ex ovo* model showing tumor regression due to Phemindole treatment at 3, 6, and 12 days. Values are mean ± SEM of three independent experiments in each case or representative of typical experiment. ^∗∗^*P* < 0.01, and ^∗∗∗^*P* < 0.001.

Furthermore, to determine whether treatment of Phemindole inhibits the growth of solid tumor mammary cancer cells *in vivo*, female BALB/c mice were used in which 4T1 mammary carcinoma cells were injected into the mammary fat pads. The injected 4T1 cells typically formed a solid tumor in the mammary fat pad, which became visible 1 week following the injection as shown in **Figure 9A**, and the volume of tumors increased till the day of sacrifice. Starting 1 week after the injection of 4T1 cells until sacrifice, the mice were injected with Phemindole (10 and 15 mg/kg body weight) i.p every alternate day for next 14 days. The overall size of the tumor in the mammary fat pad region showed a proportional morphological difference in Phemindole treated mice as compared to the untreated one (**Figure 9A**). Phemindole administration at 10 mg/kg significantly suppressed tumor growth. At the end of the experiment, tumor weight was found to be significantly lower in the mice treated with 15 mg/kg of Phemindole as compared to control subject (**Figure 9B**). Moreover, the administration of Phemindole significantly reduced the volume of tumor as compared to the control (**Figure 9C**). Furthermore, we were also interested in exploring and validating the mechanism of action of Phemindole in the *in vivo* model. It was observed that there was a significant decrease in the expression of STIM1 as evident from western blot (**Figure 9D**). Simultaneously, we have also examined the changes in expression of CHOP, and pFAK after the treatment with Phemindole (**Figure 9D**). All these findings thus cumulatively suggested that this newly synthesized indole derivative Phemindole could also effectively reduce *in vivo* solid tumor growth by lowering down STIM1 expression levels in mice models.

### Validation of Tumor Suppressive Effect Phemindole in *Ex Ovo* Model

The chick embryo model has long been used for the investigation of oncogenesis and angiogenesis (Ossowski and Reich, 1980). Interestingly, the chick embryo have served as a very useful model for the investigation of tumor cell apoptosis due to its advantages of being a natural immunodeficient host allowing easy transplantation and a suitable *in vivo* experimental system as validated by many studies (Gordon and Quigley, 1986). Chick embryos, when inoculated with 1 × 10^5^ MDAMB-231 cells onto their CAM, primary tumors were developed within 12 days (**Figure 9E**). Photographs from **Figure 9E** evidently display suppression of tumor formation on treatment with Phemindole at a dose of 10 μM.

### Comparative Assessment of *In Vivo* Hepato and Nephro Toxicity of Phemindole

**FIGURE 10:**
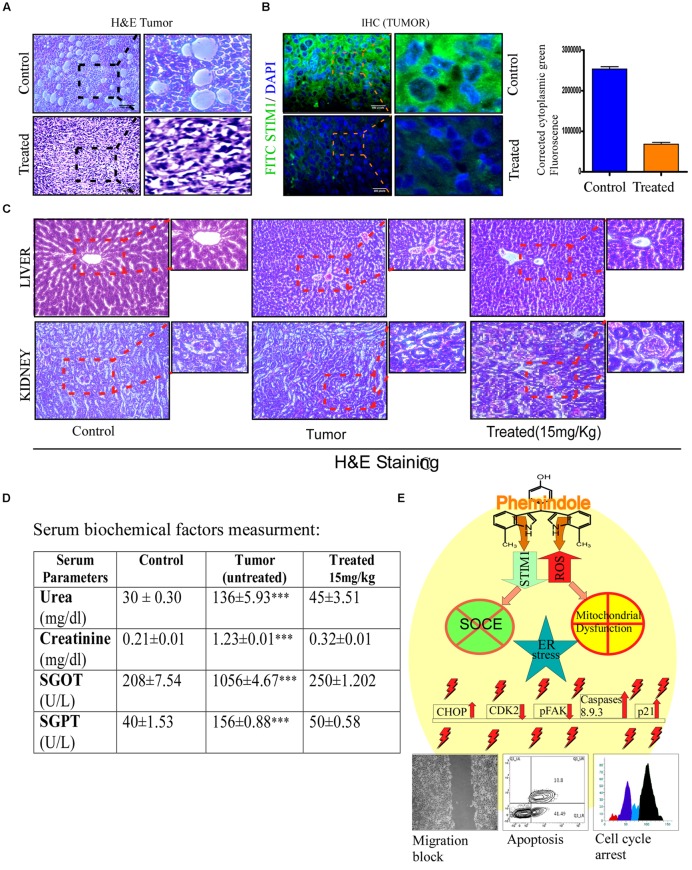
**Assessment of toxicity of Phemindole.** **(A)** 4T1 induced tumors excised from the aforementioned sets were analyzed histopathologically using eosin haematoxylin staining and phase contrast images were captured. **(B)** Immuno-histochemical analysis of the excised tumors from the untreated and Phemindole treated sets using anti-STIM1 antibody (left panel). Corrected Fluorescence intensity of STIM1 protein was quantified and represented as bar diagram (right panel). **(C)** Histological sections of liver and kidney from the animals were stained with haematoxylin and counter-stained with eosin and microscopically analyzed for histopathologically examinations of tissue toxicity like cellular damage and vacuolization. **(D)** Blood was collected after sacrifice the mice from control, untreated tumor and Phemindole treated sets and subsequent Liver function test and Kidney Function Test were performed. All measurements of three groups were repeated at least three times, Values are mean ± SEM of three independent experiments in each case or representative of typical experiment. ^∗∗∗^*P* < 0.001, **(E)** Schematic diagram depicting the detailed molecular mechanisms of antitumorogenic activity of Phemindole against triple negative breast cancer cells.

In order to analyze the tumor sections histopathologically, H&E staining was performed. It was observed that tissue sections of the control tumors revealed higher infiltration and poorly differentiated structures. No such cellular/tissue damages were observed in Phemindole treated sets (**Figure 10A**). Immuno histo Chemistry study against STIM1 antibody also revalidated our findings (**Figure 10B**). Histopathological data also showed that Phemindole delivered a complete protection from the damage produced by the tumor to the kidney and liver (**Figure 10C**). It is quite well known that Aspartate aminotranferases (SGOT), alanine aminotransferases (SGPT), and Urea, Createnine (ALP) are the enzymes found in liver and kidney which corresponds to the biochemical indices of liver function tests and kidney functions tests. Any changes in the levels of these enzymes may contribute to liver and kidney injury status. In order to evaluate whether the Phemindole caused any hepatotoxicity or nephrotoxicity the serum biochemical parameters such as SGPT, SGOT, Urea and Creatinine were measured from the serum collected from blood of both the treated and untreated sets of mice. We observed that activities of these enzymes in the serum were significantly altered in untreated tumor bearing mice sets as compared normal untreated animals. However, Phemindole provided a complete safeguard to the liver and kidney restoring to the normal level (**Figure 10D**). In conclusion, our experimental set up portrays that the Phemindole proves to be a very effective therapeutic option against TNBC cells as summarized in **Figure 10E**.

## Discussion

One of the deadliest aspects of TNBC cells is their aggressive nature and high metastatic ability thereby rendering them resistant to most commonly available therapies (Minami et al., 2011). Therefore by elucidating the critical molecular mechanisms of TNBC and targeting these mechanisms with alternative therapeutic agents may help to take significant clinical strides in the coming years. It has been acknowledged that STIM/Orai-mediated SOCE facilitates tumor growth and metastasis in different types of cancer and also plays a significant role in drug resistant breast cancer cells (Schmidt et al., 2014). Furthermore, STIM1 or Orai1 was found to be over expressed in tumor tissues when compared with pre-cancerous tissues in breast cancer patients (Yang et al., 2009). Knockdown or pharmacological inhibition of STIM1–Orai1 can thus effectively confine the growth and metastasis of breast cancer (Xie et al., 2015). Various mechanisms have been implicated in the induction of SOCE mediated apoptosis, however, none of them seemed to be significantly operative in TNBC cells thereby rendering them significantly resistant to apoptosis induced by the non-specific SOCE inhibitors. Nevertheless, SOCE inhibitor mediated apoptosis was found to be readily triggered in the TNBC cells on simultaneous activation of ER stress and ROS mediated pathway. These findings thereby suggested that the coupled action of ER stress and ROS with SOCE inhibitor may significantly enhance the anti proliferative as well as anti metastatic potential of SOCE inhibitor in TNBC cells.

In the current study we have attempted to investigate the anti-carcinogenic property of Phemindole, a synthetic analog of 3,3′di Indolyl Methane[DIM]. Phemindole was found to be a potent inducer of apoptosis in the TNBC cells. But a clear understanding of the exact molecular targets of Phemindole is required to specifically designate the exact dose of Phemindole required for chemotherapeutic use in future. The present study thus furnishes multiple evidences to confirm that Phemindole directed multiple signaling pathways culminates to apoptosis, cell cycle retardation and finally inhibition of migration of MDAMB-231 cells.

The present study demonstrated that Phemindole in a dose dependent manner induced cell death and prevented cell proliferation. Interestingly, Phemindole was found to significantly alter the level of ER calcium release in cytosol as well as suppress the SOCE activation due to ER store depletion. Several studies have already shown that alternation in SOCE was one of the major reasons for transformation of malignancy as well as apoptotic resistance phenomena in different type of premalignant cells (Prevarskaya et al., 2011). STIM1 and Orai1 are the key players of SOCE. Our observation revealed that Phemindole treatment altered SOCE by down regulating the expression of STIM1 in MDAMB-231 cells. On the other hand, the other partner protein Orai1 expression level remained unchanged in presence of Phemindole. However, the STIM1- Orai1 interaction was also turned down in plasma membrane. This type of interaction is responsible for calcium entry in response to store depletion. Our data incarnate the recent study which indicated that STIM1 also may be responsible in the development of aggressive nature of MDAMB-231 (Bergmeier et al., 2013). Other studies have also shown that knockdown of SOCE related protein significantly blocked the proliferation and migration of cancer cell (Prevarskaya et al., 2011; Bergmeier et al., 2013). In these respect our data is quite promising since Phemindole decreased STIM1 expression and induced cell death by weakening SOCE in TNBC cells. Using pharmacological Inhibitor of SOCE SKF-96365 we also revalidated the effect of Phemindole mediated SOCE inhibition in MDAMB-231 cells. Whereas, when we over expressed STIM1 protein in Phemindole treated cells, it restored SOCE and the death percentage was also reduced significantly in comparison with Phemindole treated empty vector transfected control MDAMB-231 cells. These results implied that Phemindole targets STIM1 to alter the SOCE of TNBC cells for undertaking its anti-carcinogenic activity.

However, we also found that Phemindole regulates various other mechanisms including ER stress and ROS generation. Stress in ER was produced by ER Ca^2+^ depletion due to the blocking of SOCE by Phemindole. It has been suggested that this change in ER Ca^2+^ homeostasis may cause improper protein folding in the ER Lumen and cause ER stress (Zhang and Kaufman, 2008). Consistent with this, we found that significant ER stress was induced by Phemindole in MDAMB-231 cells which was evident from the upregulation of the ER stress related marker proteins GRP78/Bip and CHOP/GADD153.

Hence, it is suggested that Phemindole which can affect both SOCE and ER stress could be a new chemotherapeutic strategy in the treatment of TNBC cells. Furthermore our findings highlighted that Phemindole treatment potentiated the ER stress mediated pathway. However, ROS scavenger NAC co-treatment with Phemindole also decreased the percentage of death cell but it cannot completely revert back the effect of Phemindole in MDAMB-231 cells. The STIM1 over expression reverted back the Phemindole-mediated SOCE alternation and cell viability. Our data thus suggested that Phemindole caused ROS generation and activated the mitochondrial apoptotic cascade and also simultaneously targeted STIM1 mediated SOCE suppression and depleted ER store leading to ER Stress-mediated apoptotic cell death in TNBC cells. Our results also highlighted that Phemindole significantly arrested cell cycle progression and retardate migration of the MDAMB-231 cells which further culminates in producing an overall anti-tumorigenic effect against TNBC cells. Our *in vitro* data were also re-evaluated by our *in vivo* results where significant tumor growth restriction was observed in both 4T1 bearing BALB/c mice models as well as in the *ex ovo* models thereby clearly highlighting the importance of Phemindole as a potential anti-tumorigenic agent.

## Conclusion

Store Operated Calcium Entry is effectively engaged in the MDAMB-231 cell growth and differentiation. Phemindole treatment attenuated SOCE and induced ER stress mediated cell death. Simultaneously Phemindole exerted ROS mediated mitochondrial membrane disruption. Coupling effect of these two pathways caused huge percentage of TNBC cell death after Phemindole treatment. To the best of our knowledge, this is the first report that Phemindole induces ER stress mediated apoptosis in TNBC cells by targeting SOCE through down-regulation of STIM1 protein. Phemindole also plays a significant role in controlling cell cycle progression and migration of TNBC cells. Thus further study with this novel molecule may help to bring about a new era in chemotherapeutic drug development.

## Author Contributions

Conceived and designed the experiments: SC and PS. Performed the experiments: SC, SG, BB, and AS. Analyzed the data: SC, SG, BB, AA, AM, and PS. Contributed reagents/materials/analysis tools: AA, AM, and PS. Wrote the paper: SC, SG, BB, and PS.

## Conflict of Interest Statement

The authors declare that the research was conducted in the absence of any commercial or financial relationships that could be construed as a potential conflict of interest.

## References

[B1] Azimi I. Roberts-Thomson S. J. Monteith G. R. (2014). Calcium influx pathways in breast cancer: opportunities for pharmacological intervention. *Br. J. Pharmacol.* 171 945–960. 10.1111/bph.12486 24460676 PMC3925034

[B2] Barboule N. Lafon C. Chadebech P. Vidal S. Valette A. (1999). Involvement of p21 in the PKC-induced regulation of the G2/M cell cycle transition. *FEBS Lett.* 444 32–37. 10.1016/S0014-5793(99)00022-8 10037143

[B3] Bergmeier W. Weidinger C. Zee I. Feske S. (2013). Emerging roles of store-operated Ca(2+) entry through STIM and ORAI proteins in immunity, hemostasis and cancer. *Channels* 7 379–391. 10.4161/chan.24302 23511024 PMC3913761

[B4] Berridge M. J. Bootman M. D. Roderick H. L. (2003). Calcium signalling: dynamics, homeostasis and remodelling. *Nat. Rev. Mol. Cell Biol.* 4 517–529. 10.1038/nrm1155 12838335

[B5] Berridge M. J. Lipp P. Bootman M. D. (2000). The versatility and universality of calcium signalling. *Nat. Rev. Mol. Cell Biol.* 1 11–21. 10.1038/35036035 11413485

[B6] Bhattacharya S. Ahir M. Patra P. Mukherjee S. Ghosh S. Mazumdar M. (2015). PEGylated-thymoquinone-nanoparticle mediated retardation of breast cancer cell migration by deregulation of cytoskeletal actin polymerization through miR-34a. *Biomaterials* 51 91–107. 10.1016/j.biomaterials.2015.01.007 25771001

[B7] Bjeldanes L. F. Kim J. Y. Grose K. R. Bartholomew J. C. Bradfield C. A. (1991). Aromatic hydrocarbon responsiveness-receptor agonists generated from indole-3-carbinol in vitro and in vivo: comparisons with 2,3,7,8-tetrachlorodibenzo-p-dioxin. *Proc. Natl. Acad. Sci. U.S.A.* 88 9543–9547. 10.1073/pnas.88.21.9543 1658785 PMC52754

[B8] Bravo R. Parra V. Gatica D. Rodriguez A. E. Torrealba N. Paredes F. (2013). Endoplasmic reticulum and the unfolded protein response: dynamics and metabolic integration. *Int. Rev. Cell Mol. Biol.* 301 215–290. 10.1016/B978-0-12-407704-1.00005-1 23317820 PMC3666557

[B9] Carrasco S. Meyer T. (2011). STIM proteins and the endoplasmic reticulum-plasma membrane junctions. *Annu. Rev. Biochem.* 80 973–1000. 10.1146/annurev-biochem-061609-165311 21548779 PMC3897197

[B10] Chen I. McDougal A. Wang F. Safe S. (1998). Aryl hydrocarbon receptor-mediated antiestrogenic and antitumorigenic activity of diindolylmethane. *Carcinogenesis* 19 1631–1639. 10.1093/carcin/19.9.1631 9771935

[B11] Chen T. Zhu J. Zhang C. Huo K. Fei Z. Jiang X. -F. (2013). Protective effects of SKF-96365, a non-specific inhibitor of SOCE, against MPP(+)-induced cytotoxicity in PC12 cells: potential role of homer1. *PLoS ONE* 8:e55601 10.1371/journal.pone.0055601 PMC3561331 23383239

[B12] De Laurentiis M. Cianniello D. Caputo R. Stanzione B. Arpino G. Cinieri S. (2010). Treatment of triple negative breast cancer (TNBC): current options and future perspectives. *Cancer Treat. Rev.* 36(Suppl. 3), S80–S86. 10.1016/s0305-7372(10)70025-6 21129616

[B13] Dejeans N. Tajeddine N. Beck R. Verrax J. Taper H. Gailly P. (2010). Endoplasmic reticulum calcium release potentiates the ER stress and cell death caused by an oxidative stress in MCF-7 cells. *Biochem. Pharmacol.* 79 1221–1230. 10.1016/j.bcp.2009.12.009 20006589

[B14] Gordon J. R. Quigley J. P. (1986). Early spontaneous metastasis in the human epidermoid carcinoma HEp3/chick embryo model: contribution of incidental colonization. *Int. J. Cancer* 38 437–444. 10.1002/ijc.2910380321 3744594

[B15] Hall J. M. Barhoover M. A. Kazmin D. McDonnell D. P. Greenlee W. F. Thomas R. S. (2010). Activation of the aryl-hydrocarbon receptor inhibits invasive and metastatic features of human breast cancer cells and promotes breast cancer cell differentiation. *Mol. Endocrinol.* 24 359–369. 10.1210/me.2009-0346 20032195 PMC2817602

[B16] Hayes D. F. (2010). Contribution of biomarkers to personalized medicine. *Breast Cancer Res.* 12(Suppl. 4):S3 10.1186/bcr2732 PMC3005723 21172087

[B17] Heath E. I. Heilbrun L. K. Li J. Vaishampayan U. Harper F. Pemberton P. (2010). A phase I dose-escalation study of oral BR-DIM (BioResponse 3,3’- Diindolylmethane) in castrate-resistant, non-metastatic prostate cancer. *Am. J. Transl. Res.* 2 402–411. 20733950 PMC2923864

[B18] Hudis C.A. Gianni L. (2011). Triple-negative breast cancer: an unmet medical need. Oncologist 16(Suppl. 1), 1–11. 10.1634/theoncologist.2011-S1-01 21278435

[B19] Koehn F. E. Carter G. T. (2005). The evolving role of natural products in drug discovery. *Nat. Rev. Drug Discov.* 4 206–220. 10.1038/nrd1657 15729362

[B20] Kondratska K. Kondratskyi A. Yassine M. Lemonnier L. Lepage G. Morabito A. (2014). Orai1 and STIM1 mediate SOCE and contribute to apoptotic resistance of pancreatic adenocarcinoma. *Biochim. Biophys. Acta* 1843 2263–2269. 10.1016/j.bbamcr.2014.02.012 24583265

[B21] Kurose K. Hoshaw-Woodard S. Adeyinka A. Lemeshow S. H. Watson P. Eng C. (2001). Genetic model of multi-step breast carcinogenesis involving the epithelium and stroma: clues to tumour–microenvironment interactions. *Hum. Mol. Genet.* 10 1907–1913. 10.1093/hmg/10.18.1907 11555627

[B22] Lax A. Soler F. Fernández-Belda F. (2006). Cytoplasmic Ca2+ signals and cellular death by apoptosis in myocardiac H9c2 cells. *Biochim. Biophys. Acta* 1763 937–947. 10.1016/j.bbamcr.2006.05.009 16887208

[B23] Li G. Zhang Z. Wang R. Ma W. Yang Y. Wei J. (2013). Suppression of STIM1 inhibits human glioblastoma cell proliferation and induces G0/G1 phase arrest. *J. Exp. Clin. Cancer Res.* 32 20–20. 10.1186/1756-9966-32-20 23578185 PMC3639102

[B24] Li W. Zhang M. Xu L. Lin D. Cai S. Zou F. (2013). The apoptosis of non-small cell lung cancer induced by cisplatin through modulation of STIM1. *Exp. Toxicol. Pathol.* 65 1073–1081. 10.1016/j.etp.2013.04.003 23714431

[B25] Liou J. Kim M. L. Heo W. D. Jones J. T. Myers J. W. Ferrell J. E. (2005). STIM is a Ca2+ sensor essential for Ca2+-store-depletion-triggered Ca2+ influx. *Curr. Biol.* 15 1235–1241. 10.1016/j.cub.2005.05.055 16005298 PMC3186072

[B26] Luo M. Guan J. -L. (2010). Focal adhesion kinase: a prominent determinant in breast cancer initiation, progression and metastasis. *Cancer Lett.* 289 127–139. 10.1016/j.canlet.2009.07.005 19643531 PMC2854647

[B27] McAndrew D. Grice D. M. Peters A. A. Davis F. M. Stewart T. Rice M. (2011). ORAI1 -mediated calcium influx in lactation and in breast cancer. *Mol. Cancer Ther.* 10 448–460. 10.1158/1535-7163.mct-10-0923 21224390

[B28] Minami C. A. Chung D. U. Chang H. R. (2011). Management options in triple-negative breast cancer. *Breast Cancer* 5 175–199. 10.4137/BCBCR.S6562 21863131 PMC3153117

[B29] Mishra B. B. Tiwari V. K. (2011). Natural products: an evolving role in future drug discovery. *Eur. J. Med. Chem.* 46 4769–4807. 10.1016/j.ejmech.2011.07.057 21889825

[B30] Mitra S. K. Hanson D. A. Schlaepfer D. D. (2005). Focal adhesion kinase: in command and control of cell motility. *Nat. Rev. Mol. Cell Biol.* 6 56–68. 10.1038/nrm1549 15688067

[B31] Nachshon-Kedmi M. Fares F. A. Yannai S. (2004). Therapeutic activity of 3,3’-diindolylmethane on prostate cancer in an in vivo model. *Prostate* 61 153–160. 10.1002/pros.20092 15305338

[B32] Ossowski L. Reich E. (1980). Experimental model for quantitative study of metastasis. *Cancer Res.* 40 2300–2309. 7190062

[B33] Pan Z. Zhao X. Brotto M. (2012). Fluorescence-based measurement of store-operated calcium entry in live cells: from cultured cancer cell to skeletal muscle fiber. *J. Vis. Exp.* 60:3415 10.3791/3415 PMC3376931 22349010

[B34] Prevarskaya N. Skryma R. Shuba Y. (2011). Calcium in tumour metastasis: new roles for known actors. *Nat. Rev. Cancer* 11 609–618. 10.1038/nrc3105 21779011

[B35] Putney J. W. Jr. (1986). A model for receptor-regulated calcium entry. *Cell Calcium* 7 1–12. 10.1016/0143-4160(86)90026-6 2420465

[B36] Safe S. Papineni S. Chintharlapalli S. (2008). Cancer chemotherapy with indole-3-carbinol, bis(3’-indolyl)methane and synthetic analogs. *Cancer Lett.* 269 326–338. 10.1016/j.canlet.2008.04.021 18501502 PMC2574232

[B37] Schmidt S. Liu G. Liu G. Yang W. Honisch S. Pantelakos S. (2014). Enhanced orai1 and STIM1 expression as well as store operated Ca(2+) entry in therapy resistant ovary carcinoma cells. *Oncotarget* 5 4799–4810. 10.18632/oncotarget.2035 25015419 PMC4148100

[B38] Selvaraj S. Sun Y. Sukumaran P. Singh B. B. (2015). Resveratrol activates autophagic cell death in prostate cancer cells via downregulation of STIM1 and the mTOR pathway. *Mol Carcinog.* 55 818–831. 10.1002/mc.22324 25917875 PMC4624064

[B39] Spassova M. A. Soboloff J. He L. P. Xu W. Dziadek M. A. Gill D. L. (2006). STIM1 has a plasma membrane role in the activation of store-operated Ca(2+) channels. *Proc. Natl. Acad. Sci. U.S.A.* 103 4040–4045. 10.1073/pnas.0510050103 16537481 PMC1449642

[B40] Stowe D. F. Camara A. K. S. (2009). Mitochondrial reactive oxygen species production in excitable cells: modulators of mitochondrial and cell function. *Antioxid. Redox Signal.* 11 1373–1414. 10.1089/ars.2008.2331 19187004 PMC2842133

[B41] Sun Y. Cui X. Wang J. Wu S. Bai Y. Wang Y. (2015). Stromal interaction molecule 1 (STIM1) silencing inhibits tumor growth and promotes cell cycle arrest and apoptosis in hypopharyngeal carcinoma. *Med. Oncol.* 32:150 10.1007/s12032-015-0608-9 25832866

[B42] Tabas I. Ron D. (2011). Integrating the mechanisms of apoptosis induced by endoplasmic reticulum stress. *Nat. Cell Biol.* 13 184–190. 10.1038/ncb0311-184 21364565 PMC3107571

[B43] Tu Y.-F. Kaipparettu B.A. Ma Y. Wong L.-J.C. (2011). Mitochondria of highly metastatic breast cancer cell line MDA-MB-231 exhibits increased autophagic properties. *Biochim. Biophys. Acta* 1807 1125–1132. 10.1016/j.bbabio.2011.04.015 21570379

[B44] Wen J. Huang Y. -C. Xiu H. -H. Shan Z. -M. Xu K. -Q. (2016). Altered expression of stromal interaction molecule (STIM)-calcium release-activated calcium channel protein (ORAI) and inositol 145-trisphosphate receptors (IP3Rs) in cancer: will they become a new battlefield for oncotherapy? *Chin. J. Cancer* 35 1–9. 10.1186/s40880-016-0094-2 27013185 PMC4807559

[B45] Xie J. Pan H. Yao J. Zhou Y. Han W. (2015). SOCE and cancer: recent progress and new perspectives. *Int. J. Cancer* 138 2067–2077. 10.1002/ijc.29840 26355642 PMC4764496

[B46] Yang S. Zhang J. J. Huang X. -Y. (2009). Orai1 and STIM1 are critical for breast tumor cell migration and metastasis. *Cancer Cell* 15 124–134. 10.1016/j.ccr.2008.12.019 19185847

[B47] Zhang K. Kaufman R. J. (2008). From endoplasmic-reticulum stress to the inflammatory response. *Nature* 454 455–462. 10.1038/nature07203 18650916 PMC2727659

[B48] Zijlstra A. Mellor R. Panzarella G. Aimes R. T. Hooper J. D. Marchenko N. D. (2002). A quantitative analysis of rate-limiting steps in the metastatic cascade using human-specific real-time polymerase chain reaction. *Cancer Res.* 62 7083–7092. 12460930

